# The Link between Hypersensitivity Syndrome Reaction Development and Human Herpes Virus-6 Reactivation

**DOI:** 10.1155/2012/723062

**Published:** 2012-05-16

**Authors:** Joshua C. Pritchett, Radu M. Nanau, Manuela G. Neuman

**Affiliations:** ^1^HHV-6 Foundation, Santa Barbara, CA 93108, USA; ^2^Department of Pharmacology and Toxicology, University of Toronto and In Vitro Drug Safety and Biotechnology, Toronto, ON, Canada M5G 1L7

## Abstract

*Background*. There are challenges in the clinical diagnosis of drug-induced injury and in obtaining information on the reactivation of human herpes viruses (HHV) during idiosyncratic adverse drug reactions. *Objectives*. (i) To develop a unified list of drugs incriminated in drug-induced hepatotoxicity and severe cutaneous reactions, in which drug hypersensitivity leads to HHV-6 reactivation and further complication of therapy and recovery and (ii) to supplement the already available data on reporting frequencies of liver- or skin-induced cases with knowledge of individual case reports, including HHV-6 reactivation and briefly introducing chromosomally integrated HHV-6. *Data Sources and Extraction*. Drugs identified as causes of (i) idiosyncratic reactions, (ii) drug-induced hypersensitivity, drug-induced hepatotoxicity, acute liver failure, and Stevens-Johnson syndrome, and (iii) human herpes virus reactivation in PubMed since 1997 have been collected and discussed. *Results*. Data presented in this paper show that HHV-6 reactivation is associated with more severe organ involvement and a prolonged course of disease. *Conclusion*. This analysis of HHV-6 reactivation associated with drug-induced severe cutaneous reactions and hepatotoxicity will aid in causality assessment and clinical diagnosis of possible life-threatening events and will provide a basis for further patient characterization and therapy.

## 1. Introduction

A hypersensitivity reaction (HSR) is a host-dependent idiosyncratic adverse drug reaction (ADR) that cannot be predicted by the dose, frequency, or length of the treatment. Furthermore, animal models cannot predict an HSR. A “true” HSR is defined by the triad of fever, rash, and internal organ involvement [1–6]. HSRs occur with an incidence of 1 in 1000 to 1 in 10000 drug exposures, with a mortality rate approaching 10% [5–8]. Symptoms of HSR can range from general manifestations, such as morbiliform rash, urticaria, angioedema, fever, malaise, anaphylaxis, bronchospasm, and erythema multiforme, to severe cutaneous adverse reactions (SCAR) such as drug-induced hypersensitivity syndrome (DIHS)/drug reaction with eosinophilia and systemic symptoms (DRESS), and Stevens-Johnson syndrome (SJS)/toxic epidermal necrolysis (TEN) [2, 4–7, 9–11]. Cutaneous eruptions are reported in the vast majority of DIHS/DRESS cases, often presenting as maculopapular rash or generalized erythematous rash [7, 8]. The internal organ most often affected is the liver, with drug-induced liver injury (DILI) presenting as anomalies in liver function tests or the presence of hepatomegaly. Aspartate aminotransferase (AST) and alanine aminotransferase (ALT) were found to be increased an average of 9-10-fold above the upper normal limits in a literature review of 172 DRESS cases [7]. The presence of serum bilirubin raised >3 times over the upper limit of normal along with aminotransferase elevations is associated with a more severe set of symptoms than isolated aminotransferase abnormalities alone, an observation known as Hy's Law [12, 13]. Other internal organs, such as kidneys, are less often affected [4, 7]. Hypereosinophilia is the third most common symptom of DIHS/DRESS [7]. Skin rash, liver involvement, high-grade fever, hypereosinophilia, lymphadenopathy, and the increased presence of atypical lymphocytes are symptoms associated with DIHS/DRESS cases classified as “probable/definite” based on the RegiSCAR scoring system [7].

Maculopapular rash, erythema multiforme, exfoliative dermatitis, acute generalized exanthematous pustular dermatosis-like eruptions, and erythroderma are types of cutaneous manifestations associated with DIHS, while mucosal involvement is rare. On the other hand, skin and mucosal involvement characterizes SJS/TEN [8]. A further distinction between DIHS/DRESS and SJS/TEN is the time to onset of symptoms, which is often delayed (4-5 weeks) in the former, while it occurs in the early stages of drug exposure (approximately 3 weeks) in the latter [8].

Carbamazepine was the drug most often associated with DRESS among 44 drugs linked to this reaction in 172 cases reported over a period of 12 years [7]. Anticonvulsants such as carbamazepine, phenytoin, phenobarbital, lamotrigine, zonisamide, and sodium valproate are the class of drugs most often related to DIHS/DRESS. Other pharmaceutical agents, including allopurinol, nonsteroidal anti-inflammatory drugs, chlormezanone, aminopenicillins, cephalosporins, quinolones cycline antibiotics, and antiretrovirals, as well as anti-infective sulfonamides, have also been implicated [6–8, 11, 14–16].

SCAR development results in patient hospitalization, and the culprit drug is interrupted on the first day of hospitalization. DILI is often diagnosed during the course of the disease. Corticosteroids are the treatment most often employed. The mean time to recovery was 6.4 weeks in 172 DRESS cases, while death was recorded in 9 (5.2%) patients. Death was connected with a more severe course of reaction, particularly observed in older patients, and often due to liver failure [7].

While the precise mechanism of HSR is unknown, various theories involve the interplay between metabolic and immunologic factors [2, 4, 6], genetic predisposition, and, more recently, infection with human herpes viruses (HHV), particularly HHV-6 [8].

Drug metabolites bind to cellular macromolecules such as proteins, creating covalent adducts that can serve as antigenic stimuli for the immune system [4, 6]. Several genetic factors are believed to predispose certain individuals to this type of adverse reaction. The subset of the population that is susceptible to HSRs carries defects in drug detoxifying pathways (e.g., epoxide hydrolase), such that a greater amount of a reactive drug metabolite is produced than that which can be detoxified [2, 4, 6]. Additional genetic predispositions that render certain individuals susceptible to HSRs include human leukocyte antigen (HLA) alleles, which are part of the major histocompatibility complex (discussed by Neuman et al. [17]).

Reactivation of latent viral infections has been linked to the development of more severe HSR symptoms, particularly in the context of DIHS/DRESS. For example, HHV-6 reactivation was associated with more severe organ involvement and a prolonged course of disease in 62 of 100 DIHS patients, compared with the remaining 38 patients who did not experience HHV-6 reactivation [18]. In the same study, all five deaths and ten cases of renal failure were observed among the 62 patients with HHV-6 DNA detected in the serum [18].

HHV-6 is a lymphotropic DNA virus belonging to the *Betaherpesviridae* subfamily [19, 20]. Two genetic variants of HHV-6 exist, HHV-6A and HHV-6B. While little is known about HHV-6A, HHV-6B infection often occurs during the first 2 years of life and is associated with acute febrile illness in young children and exanthem subitum in infants [19–26]. HHV-6 infection can be accompanied by convulsions and febrile status epilepticus. HHV-6 has been increasingly recognized as a cause of hepatitis and liver failure, as well as increased graft rejection and consequent decreased patient survival [20–26]. The rate of infection approaches 100% in individuals 2-3 years of age [21].

Following the initial infection, HHV-6B remains present in a latent phase, and infection can be reactivated during episodes of immunosuppression [20, 22, 24, 26]. Inherited forms of both HHV-6A and HHV-6B have been shown to integrate in chromosomes. This condition, known as chromosomally integrated HHV-6 (CIHHV-6), occurs in 0.8% of control populations but is found in approximately 2% of patient populations in Europe, United States, and the United Kingdom, with a lower rate in Japan. CIHHV-6 is characterized by the complete integration of the HHV-6 genome into the host germ line genome, such that CIHHV-6 DNA is found in every nucleated cell in the body [24, 27]. Persistently high viral copy numbers in whole blood or serum are observed in individuals with this condition [28, 29]. The viral genome of CIHHV-6 is transmitted vertically in the germline in a Mendelian manner [27]. Approximately 1% of newborns are diagnosed with congenital HHV-6, all of which are infected with CIHHV-6, directly or indirectly. In a sample of 43 infants with congenital infection, the majority (86.0%) were born with inherited CIHHV-6, while the remaining (14.0%) were not born with CIHHV-6 but became infected with HHV-6 transplacentally from their CIHHV-6-positive mothers [30].

The cellular DNA damage machinery responds to virus infection and the foreign genomes that accumulate in the nuclei of infected cells. Many DNA viruses have been shown to manipulate the cellular DNA damage response pathways in order to create environments conducive to their own replication. Some cellular factors are activated during infection while others are inactivated [31].

Arbuckle et al. showed that HHV-6 DNA integrates specifically and efficiently into the telomere sequences of chromosomes. The integrated virus can be chemically activated with trichostatin A, a stimulatory compound known to reactivate latent herpesviruses [32].

Active HHV-6 replication is determined by the presence of HHV-6 DNA in serum, plasma, or cerebrospinal fluid, or by reverse-transcriptase polymerase chain reaction (RT-PCR) in peripheral blood mononuclear cells (PBMC). RT-PCR is the sole technique that allows active HHV-6 replication to be differentiated from inactive CIHHV-6 [33]. It is important that active HHV-6 replication is differentiated from CIHHV-6 [34].

The present paper reviews recently published case reports of SCAR (DIHS/DRESS and SJS/TEN) and DILI developed secondary to drug exposure, with a particular focus on the role of HHV-6 reactivation and CIHHV-6 presence in the clinical outcome of the reaction. Our principal aim is to address the role of HHV-6 reactivation in the course of HSRs, particularly in DILI and DIHS/DRESS. In addition, we will focus on the complex cellular responses triggered by HHV-6 reactivation.

## 2. Materials and Methods

Studies discussed in this paper were selected based on a PubMed search of English language papers published in the last 15 years (1997–2011) using keywords such as “HHV-6 and hypersensitivity,” “HHV-6 and DIHS,” “HHV-6 and DRESS,” “HHV-6 and SJS,” “HHV-6 and TEN,” “HHV-6 reactivation and drug,” “HHV-6 and drug rash,” and “chromosomally integrated HHV-6.” Case reports presented deal largely with anticonvulsants and included HHV-6 reactivation. The selected case reports are part of a larger database of HSR cases with HHV-6 reactivation. In addition, our laboratory has studied HSRs and their mechanisms for the last 25 years, and we discuss the cases based on our experience related to the laboratory and clinical manifestations.

## 3. Results

### 3.1. HSR and HHV-6 Reactivation

Following initial infection in early childhood, HHV-6 continues to exist in a latent phase in the body. Consequently, HHV-6 DNA can be measured in different body compartments in HHV-6-positive individuals without active infection. Trace amounts of HHV-6 viral DNA were also detected by real-time quantitative PCR in 91 (26.8%) of 339 pediatric patients diagnosed and treated for acute lymphoblastic leukemia [34]. Low rates of HHV-6B DNA were detected in whole blood (8.0%), PBMCs (16.5%), and polymorphonuclear leukocytes (10.5%) belonging to 200 volunteers [54]. HHV-6A DNA was not observed in this sample. The mean observed HHV-6B viral loads were 81 copies/10^6^ cells in whole blood, 62 copies/10^6^ cells in PBMCs, and 34.5 copies/10^6^ cells in polymorphonuclear leukocytes. Based on these findings, Géraudie et al. classify healthy individuals as having HHV-6B viral loads below 100 copies/10^6^ cells [54]. HHV-6-DNA was positive in only 27 (39.1%) blood samples from 69 children undergoing elective tonsillectomy for moderate tonsillar hyperplasia or recurrent streptococcal infection without evidence of acute HHV-6 infection, while evidence of HHV-6 DNA was found in 100% of tonsil samples in the same population [55].

The common features of DIHS identified in a sample of 7 patients were high fever (≥39°C) in all patients, facial edema in all patients, diffuse lymphadenopathy in 5 (71.4%) patients, hypereosinophilia (>0.5 × 10^9^/L) in 4 (57.1%) patients, atypical circulating lymphocytes in 4 (57.1%) patients, ALT elevated >3 times the reference level in 5 (71.4%) patients, and hypogammaglobulinemia in 4 (57.1%) patients [14]. The onset of DRESS took place after a mean period of 25 days (5 days–6 weeks) of therapy with the incriminated drug [14]. Similar findings are reported in other cohorts of DIHS patients [15, 56].

Several studies suggest that HHV-6 reactivation can only occur in susceptible individuals under conditions of transient immunosuppression, such as those transiently associated with the onset of DIHS [56, 57]. Lower serum IgG levels were observed in 10 adult anticonvulsant-induced HSR patients (mean 745 mg/dL) compared to 15 controls (*P* < 0.001). Serum IgG levels continued to decrease for several days after the drug was discontinued [57]. Similarly, circulating levels of B cells were also decreased in DIHS patients, compared to controls [15, 57]. In susceptible individuals, continuous therapy with anticonvulsants, which have been associated with DIHS, can lead to decreases in B cells and subsequent hypogammaglobulinemia. Drug-induced hypogammaglobulinemia could account for the delay between DIHS onset and HHV-6 [57]. Moreover, HHV-6 reactivation may occur at the same time, but it cannot always be detected in blood, since it takes 2–4 weeks for antibodies to appear after infection or reactivation. Because decreases in circulating IgG and B cell levels are not observed with other hypersensitivity conditions such as SJS/TEN, depressed levels of IgG and B cells can be used as biomarkers for the onset of DIHS [57].

HHV-6 reactivation may be partially explained by the immunosuppressive effects of DIHS-related drugs. The magnitude of inflammation is often proportional to the clinical manifestations, while hypogammaglobulinemia and HHV-6 reactivation worsen the clinical course of the disease [58]. Hypogammaglobulinemia is thus an early symptom of severe DIHS.

A more detailed description of disease progression is presented in case reports, some of which are summarized in Table 1 [35–52]. Fever was present in all patients described in these reports. Edema, predominantly on the face, was observed with a relatively high frequency [35, 37, 39–44, 47–49, 52], as were lymphadenopathy [35, 36, 39, 41–43, 45, 46, 49–51], hypereosinophilia [35–40, 42–52], and atypical lymphocytes [35, 36, 38–40, 42, 44, 46, 47, 52]. Internal organ involvement manifested itself largely as liver dysfunction, with elevated levels of liver enzymes [35–46, 48–50, 52] and cholestatic hepatitis with hepatocellular insufficiency [51]. Kidney failure was observed in a 75-year-old lamotrigine patient [51]. Multiple organ failure was observed in a couple of studies [47, 51], one of which resulted in death [47]. Hypogammaglobulinemia, another symptom of DIHS, was observed in a number of studies as well [35, 38, 39, 45, 49].

The offensive drug is immediately interrupted in DIHS patients, and treatment with prednisolone is often used to reverse the condition [15]. Symptoms begin to improve gradually, but both fever and skin manifestations often relapse approximately 2-3 weeks after the onset of DIHS. There is a delay until antibodies to the virus are generated. Therefore, there is a delay until the peak of the infection is detected. Symptoms relapse coincides with detection of HHV-6 reactivation, measured by circulating anti-HHV-6 antibodies and HHV-6 DNA [15, 56].

Anti-HHV-6 IgG levels are elevated after 3 weeks following the onset of the reaction (time of drug withdrawal), but not after 2 weeks [58, 59]. Increases in HHV-6 DNA in PBMCs were found to precede the increases in anti-HHV-6 IgG levels in anticonvulsant HSR patients and were absent in controls [57]. The presence of HHV-6 DNA in PBMCs was found to correlate with the magnitude of anti-HHV-6 antibody titers (*P* = 0.0002) [60]. Intermittent detection of HHV-6 DNA in PBMCs was not indicative of illness, such that HHV-6 DNA can be found in the PBMCs at low levels in immunocompetent children, with and without symptoms [60].

Significant increases in anti-HHV-6 IgG titers were noted in all 6 patients included in a study. HHV-6 infection was confirmed in all 6 patients by PCR in PBMCs. HHV-6 reactivation was observed between 17 and 27 days after the onset of the reaction [56].

The presence of HHV-6 DNA in plasma or serum is evidence of active HHV-6 infection. In addition, the presence of serum anti-HHV-6 IgM and increases in the levels of anti-HHV-6 IgG levels are used to differentiate primary HHV-6 infection (i.e., seroconversion) from HHV-6 reactivation. Serum anti-HHV-6 IgG was detected in 7 DRESS patients analyzed, with serum anti-HHV-6 IgM detected in only 4 (57.1%) patients. Seroconversion was diagnosed in 2 patients, in which serum anti-HHV-6 IgM titers were initially undetectable and became detectable in subsequent serum samples, coinciding with increases in anti-HHV-6 IgG titers [14]. Seroconversion is described in detail in a 56-year-old male patient with aromatic anticonvulsant-induced HSR [47].

Aside from the presentation of fever and cutaneous symptoms, other features of HHV-6 reactivation can include lymphadenopathy, hepatosplenomegaly, encephalitis caused by the virus, and severe lymphocytopenia [56].

HHV-6 replication in hepatocytes led to severe hepatitis, associated with elevated enzyme levels, in an 18-year-old female [25]. Anti-HHV-6 antibody titers were elevated, as were HHV-6 DNA levels [25]. Higher odds of detecting HHV-6 DNA in PBMCs and liver biopsies were found in cases of fulminant hepatic failure or acute decompensation of chronic liver disease, compared to chronic liver disease (*P* = 0.02), in a sample of 23 children (median age 24 months) with acute liver failure. Similarly, higher HHV-6 DNA levels were found in cases of fulminant hepatic failure or acute decompensation of chronic liver disease, compared to chronic liver disease cases (*P* = 0.0043). These findings suggest that HHV-6 infection may cause liver failure in children [20].

Tumor necrosis factor- (TNF) *α* and interleukin- (IL) 6 levels were initially elevated prior to HHV-6 reactivation in 4 (66.7%) of 7 patients in a study. Although initially high, IL-6 levels decreased just prior to HHV-6 reactivation. IL-6 levels were once again elevated after HHV-6 infection in 5 (83.3%) of 6 patients [56]. IL-6 and TNF-*α* are produced primarily by monocytes and macrophages, the likely reservoir cells of latent HHV-6. Yoshikawa et al. propose that HHV-6 reactivation induces cytokine synthesis, which in turn modulate clinical manifestations [56]. These cytokines likely play an important role in viral reactivation, yet the low number of patients analyzed in this study makes a definite answer impossible [56]. Serum levels of IL-5, interferon (IFN)-*γ*, and eosinophil cationic protein were increased on day 29 and decreased on day 39 during an episode of carbamazepine DIHS. IL-6 was also increased at this time [58].

An interesting case of drug sensitization was observed in a patient who developed symptoms of DIHS within a few days of being exposed to the antibiotic cefaclor. This patient was previously exposed to cefaclor during an episode of carbamazepine-induced DIHS [58]. Slight increases in IL-5, IL-6, IL-10, IFN-*γ*, and eosinophil cationic protein were elevated in sera on day 6 of cefaclor DIHS [58]. A drug-induced lymphocyte stimulation test using PBMCs showed that the patient was sensitized to carbamazepine during the first episode, but not to cefaclor. Results of the test showed sensitivity to cefaclor during the second episode [58]. During the first episode, carbamazepine-induced HSR was diagnosed 17 days after the start of carbamazepine, consistent with DIHS. Also, symptoms persisted after the drug was interrupted, and the patient responded to pulsed intravenous (IV) methylprednisolone. In contrast, the patient developed the symptoms much quicker during the second episode, during which time the symptoms were milder and disappeared faster after interruption of cefaclor. The involvement of HHV-6 during the first episode was established with anti-HHV-6 IgG titers and HHV-6 DNA. Absence of HHV-6 reactivation could explain the milder symptoms during the second episode [58]. The magnitude of inflammatory cytokine increase was higher, and eosinophilia was more pronounced during the more severe carbamazepine-induced DIHS, prompting Aihara et al. to argue that HSR is mediated by both elevated inflammatory cytokine levels and activated eosinophils [58].

It is also possible that corticosteroids administered as initial treatment for drug toxicity are involved in the reactivation of HHV-6, Epstein-Barr virus (EBV), and cytomegalovirus (CMV), which became active in the later stage of the disease [15].

Anti-HHV-6 IgG titers were elevated in 7 DIHS patients 21–38 days after the onset of the reaction. Simultaneous elevation in anti-HHV-7 IgG was observed in 6 patients. An elevation in anti-CMV IgG was observed in all patients 35–54 days after the onset of the reaction. Only 2 patients experienced an elevation in anti-EBV IgG, 37 and 48 days after the onset of the reaction. Antibodies to herpes simplex virus, varicella-zoster virus, human parvovirus B19, rubella, and measles were not changed [15]. Viral DNA was detected in whole blood in all patients for whom anti-viral IgG was observed, and in serum for 6 of 7 HHV-6 patients, 5 of 6 HHV-7 patients, and all CMV and EBV patients. Rises in CMV viral loads followed rises in HHV-6 viral loads by 10–21 days [15]. Multiple viral reactivations in a single patient are reported in another study as well [45].

HHV-6 and/or HHV-7 often reactivate first, followed by CMV and/or EBV [15]. There appears to be a correlation between relapse of symptoms and detection of increased viral loads for the virus that gets reactivated first (i.e., HHV-6 or HHV-7). Subsequent reactivation of CMV and EBV could be asymptomatic [15]. Further immune system perturbations brought about by the initial HHV reactivation are believed to be triggers for subsequent reactivation of other HHVs [15].

### 3.2. Chromosomally Integrated HHV-6

CIHHV-6 was described and quantified in healthy blood donors and various hospital populations (e.g., organ transplant recipients and immunosuppressed patients) [28, 61–67]. The mean viral loads in whole blood ranged from 10^6^ copies/mL to 10^7^ copies/mL [28, 62, 64].

Vertical transmission of inherited chromosomal integration is well documented [61, 68]. In addition, there are reports of CIHHV-6 acquisition through bone marrow transplant, followed by the presence of HHV-6 in every cell derived from hematopoietic stem cells [24, 69–71].

It is not known if CIHHV-6 can activate from its integrated state *in vivo*, but several lines of evidence suggest that this is a significant possibility. Integrated HHV-6 has been shown to be activated *in vitro *[32] using histone deacetylase inhibitors and other compounds known to reactivate herpesviruses from latency [27]. As previously discussed, transmission of CIHHV-6 from infected mothers to their non-CIHHV-6 infants, via the placenta, is another indicator that CIHHV-6 may be activated from its integrated state *in vivo* [30]. Children suspected of encephalitis in the United Kingdom had a four times greater rate of CIHHV-6 than the general population [72], and liver transplant patients with CIHHV-6 have higher rates of graft rejections and opportunistic infections [62]. Marek's Disease virus, a herpesvirus associated with tumors in chickens, can reactivate from its integrated state *in vitro* [27]. If specific drugs can cause integrated virus to activate, then individuals with CIHHV-6 may be at an increased risk for drug-induced HSR.

Only a few cases of HSR in CIHHV-6 patients have been reported. Watanabe et al. report the case of a 47-year-old Japanese male with fever (38.8°C) and generalized erythematous rash 78 days after carbamazepine exposure [73]. Mild hepatotoxicity (ALT 70 IU/L, AST 73 IU/L, *γ*-glutamyl transpeptidase 129 IU/L, and lactate dehydrogenase 686 U/L) and hypogammaglobulinemia (IgG 609 mg/dL, IgA 34 mg/dL and IgM 27 mg/dL) were measured. An abdominal skin biopsy revealed hydropic and vacuolar degeneration of epidermal basal cells, lymphocytic infiltration in the epidermis, and a dense upper dermal infiltrate consisting mainly of mononuclear cells [73]. Anti-HHV-6 IgG titers were increased 128-fold 6 weeks after the reaction onset, while anti-HHV-6 IgM titers remained unchanged throughout the course of the reaction, pointing towards HHV-6 reactivation. HHV-6 DNA was persistently high in serum (>10000 copies/mL) and whole blood. Fluorescent *in situ* hybridization revealed CIHHV-6 on chromosome 1q44 [73].

HHV-6 DNA levels in primary infection and acute reactivation are typically below 5.5 × 10^5^ copies [27]. Of interest, the only cases reported of viral loads above this level (or in the same range as individuals with CIHHV-6) appear to be those with graft-versus-host disease or DIHS, two conditions that have very similar symptoms and courses [18, 27, 74].

## 4. Discussion

In the present paper, we review cases of HSR associated with HHV-6 reactivation. In the vast majority of patients, HHV-6 infection occurs during infancy or early childhood [60]. Following resolution of the primary infection, the virus remains latent in salivary glands, PBMCs, and the central nervous system. HHV-6 reactivates in immunosuppressed individuals and is associated with fever, rash, encephalitis, bone marrow suppression, and transplanted organ rejection. Primary HHV-6 infection can be identified by the presence of HHV-6 DNA in the serum, plasma, or cerebrospinal fluid, or reverse transcription PCR in the absence of elevated antibody titers [60]. Reactivation is marked by detection of both viral DNA and elevated anti-HHV-6 antibody titers [60]. As anti-HHV-6 IgG levels increase during the course of the reaction, anti-HHV-6 IgM levels remain constant around <1 : 20 in patients with HHV-6 reactivation [42, 75].

HSRs seem to occur with almost a thousandfold higher incidence in AIDS patients compared to immunocompetent individuals exposed to the same medication, as there is a much higher exposure to drugs in these patients, as well as a much higher incidence of viral infections [76, 77]. Aside from a higher incidence of HSR, HIV positivity can also lead to more severe symptoms, compared to HIV-negative patients [77]. In addition, an *in vitro* study showed increased DNA viral replication in PBMCs belonging to DIHS patients that were depleted for natural killer cells (NK) [78]. These findings point toward an extensive immune barrier aimed against DIHS development, while immune defects in components of these pathways can explain why only a relatively small amount of the population exposed to a drug will develop the reaction.

Interestingly, Kano et al. suggest that HHV-6 reactivation is not a consequence, but rather a prerequisite for anticonvulsant HSR [57]. The expansion of CD4^+^ T cells and CD8^+^ T cells in response to HHV-6 reactivation seems to be of extreme importance in the development of multiple organ failure during the course of anticonvulsant HSR. From their immunological findings, Kano et al. believe that continuous anticonvulsant therapy leads to transient immune dysfunction, marked by decreased IgG production [57]. While T cells protect against reactivation of viruses from a latent stage, antibodies prevent the dissemination of reactivating lytic virus. Therefore, DIHS may occur when transient drops in B cell counts and immunoglobulin production allow HHV-6 to be reactivated from latency, in conjunction with the presence of drug-specific T cells. The likely explanation for the lag between start of anticonvulsant therapy and onset of disease is that time is required for immunoglobulin levels to drop below a certain threshold [57].

An *in vitro* study using the human T cell line MT4 found that therapeutic doses of both sodium valproate and carbamazepine led to enhanced HHV-6B replication in a dose-independent manner, whereas phenytoin and sulfasalazine had no effects on HHV-6B replication or cell proliferation [79]. *In vivo*, there have been reports of phenytoin and sulfonamide antibiotics leading to HHV-6 reactivation in DIHS patients [42, 43, 47, 52].

As such, while widespread, HHV-6 infection is not encountered in all DIHS cases. For example, HHV-6 DNA was detected by PCR in blood or serum in a 25-year-old female treated with phenobarbital, while it was absent in a 21-year-old female treated with phenytoin [16]. Matsuda et al. describe 2 cases of carbamazepine-induced DIHS in which no viral reactivation was observed [53]. Symptoms, including fever, skin eruptions, and liver involvement, occurred between 22 days and 4 weeks after carbamazepine initiation in the two patients, which is consistent with DIHS. Furthermore, eosinophilia and a drop in white blood cell count were observed in both patients [53].

HHV-6 reactivation typically occurs 2-3 weeks after the development of rash in DIHS patients [8, 76]. Evidence of this are elevated HHV-6 IgG titers and plasma HHV-6 DNA [76]. HHV-6 reactivation is often accompanied by relapse of fever and hepatitis [8]. A cascade of viral reactivation can then be started, with HHV-6, EBV, and/or HHV-7 at the top, followed, with some delay, by other herpes viruses, particularly CMV. Reactivation of these viruses is followed by development or exacerbation of clinical symptoms of DIHS, including various organ failures [8, 76, 80]. Of particular interest is CMV reactivation, which can be followed by transient fever, skin rash, myocarditis, pneumonia, or gastrointestinal bleeding [8].

Clinical symptoms (fever, hepatitis, and/or skin rash) correspond to the appearance of detectable HHV-6 DNA levels. Fever and/or hepatitis are the most common clinical features of HHV-6 reactivation in DIHS patients. Based on these findings, HHV-6 reactivation leads to a more severe course of disease in patients with DIHS [18]. HHV-6 reactivation is associated with more severe organ involvement and a prolonged course of the disease in patients with drug rash and systemic symptoms, compared with the remaining patients who did not experience HHV-6 reactivation [18]. Significant detection of HHV-6 DNA corresponds to the presence of anti-HHV-6 IgG titers, and there is a positive correlation between HHV-6 DNA detection and the development of fever and hepatitis [18].

While HHV-6 reactivation is a common feature of DIHS/DRESS, HHV-6 DNA was not detected in 15 patients with generalized morbilliform/maculopapular drug reactions [81]. HHV-7, CMV, or EBV DNA was not detected in any of the patients included in this study. *In situ* hybridization revealed HHV-6 mRNA in a small subset of infiltrating mononuclear cells around blood vessels and appendages in DIHS patients only [81]. Similarly, only 1 (25.0%) of 4 SJS/TEN patients for whom data was available showed an increase in anti-HHV-6 IgG titers from 1 : 10 to 1 : 1280 [52]. Aside from a lower likelihood of HHV-6 reactivation, SJS/TEN is marked by a quicker onset of reaction than DIHS/DRESS (2-3 weeks versus a mean 34 days) [52]. However, fever (>38.5°C), leukocytosis, eosinophilia, and atypical lymphocytosis were common features of both SJS/TEN and DIHS/DRESS, whereas liver dysfunction was observed predominantly in SJS/TEN patients and lymph node enlargement was observed predominantly in DIHS/DRESS patients. Treatments for SJS/TEN included systemic corticosteroids in 7 (87.5%) patients and IV immunoglobulin in 1 (12.5%) patient [52].

Furthermore, Teraki et al. warn that some of the clinical features of anticonvulsant-induced SJS/TEN may differ from SJS/TEN induced by other drugs, such as a more delayed onset of symptoms in the former category [52]. Additionally, the rate of hepatic dysfunction and hematological anomalies was also higher than that described with SJS/TEN induced by other drugs [52]. HHV-6 reactivation and atypical lymphocytosis were observed in DIHS patients predominantly, compared to SJS/TEN patients. The precise morphological nature of the skin reaction is another criterion used to differentiate DIHS from SJS/TEN. These findings should be interpreted with care due to the low sample size [52].

Carbamazepine was associated with 12 (52.2%) of 23 anticonvulsant HSR cases reported in a cohort study. All patients developed fever and pruritus. Maculopapular exanthematous eruptions were observed in 15 patients, erythroderma in 11 patients, exfoliative dermatitis in 3 patients, bullous eruption in 1 patients, urticaria in 1 patient, and purpura (vasculitis) in 2 patients [39]. There was no relationship between the drug used and the length or severity of the reaction. In addition to the skin, 20 (87.0%) patients had at least one other organ affected by the reaction, while 8 (34.8%) patients had at least two other organs affected by the reaction. DILI was observed in 12 (52.2%) patients, and kidney involvement was observed in 8 (34.8%) patients. Other affected organs include the spleen (splenomegaly), the lung, the thyroid (hypothyroidism), the heart, and the pancreas [39].

Drug-drug interactions are particularly problematic when several pharmaceutical agents capable of inducting HSR are administered simultaneously. For example, Conilleau et al. report the case of a child exposed intermittently to two anticonvulsants prior to developing DIHS [48]. Despite ethosuximide being interrupted prior to hospitalization and sodium valproate being associated more often with DIHS, it appears that the reaction was caused by the combination of these two structurally unrelated anticovulsants, as a patch test was positive for both ethosuximide and sodium valproate [48]. In a separate case, an infant that was exposed to carbamazepine, phenytoin, and phenobarbital presented with initial HHV-6 infection. Despite the patient being diagnosed with phenobarbital hypersensitivity [43], all three of these aromatic antiepileptic drugs have the potential to cause HSR [2]. Our group also describes patients susceptible to multiple antiepileptics [17].

Minocycline-induced DIHS with EBV reactivation was reported in a 24-year-old African American female. The presence of HHV-6 was not assessed in this study [82]. HHV-6 reactivation was observed in two patients with sulfasalazine severe hypersensitivity [83]. Sequential activation of HHV-6, HHV-7, herpes simplex virus, and CMV was observed in a 46-year-old male who developed DIHS after being exposed to cyanamide, a drug used to control alcoholism [84]. HHV-6 reactivation was observed in 2 elderly patients with allopurinol DIHS [26, 79], and EBV reactivation was observed in a 40-year-old black male with allopurinol DIHS and pancreatitis [85]. The cause of the reaction was unknown in an 18-year-old female with SJS/TEN exposed to valacyclovir, promethazine, synthroid, belladonna, and orthotricycline [26]. Cacoub et al. provide a comprehensive list of 44 drugs associated with DIHS/DRESS [7].

In addition to anticonvulsants, patients can be exposed to additional medication for the treatment of comorbid diseases, opportunistic infections, or even for the management of HSR symptoms (e.g., antibiotics or nonsteroidal inflammatory drugs administered for the treatment of fever). Furthermore, epilepsy patients are often exposed to more than one anticonvulsant, most of which are capable of causing an HSR. While medication is interrupted at the time of HSR diagnosis and the patient is encouraged to avoid rechallenge with the drug that was deemed to have initiated the reaction, it is still important that all medication is considered and tested individually for its capacity to initiate an HSR. Investigators seldom check to see if there is an additive effect between the incriminated anticonvulsant and other medication, or if the anticonvulsant caused the reaction on its own. Also, it is unlikely that HHV-6 reactivation is measured upon admission. As a result, it is difficult to establish whether the drug or the virus initiates the reaction. Kano et al. argue that HHV-6 reactivation creates an environment that favors DIHS development [57]. The same investigators report the case of a 46-year-old woman who developed DIHS 4.5 months after initiating therapy with zonisamide and corticosteroids [80]. Since the reaction occurred well outside the time frame characteristic of DIHS and since reactivation of herpes viruses was not measured, it is possible that this phenomenon could have created the conditions necessary for DIHS to occur in this patient with long-term exposure to anticonvulsant therapy [80].

The presence of proinflammatory cytokines during DILI and SCAR is supported by findings that a significantly higher number of eosinophils was found both in the blood and tissue of patients with a drug-induced maculopapular exanthems, compared to control subjects with normal skin and skin from patients with psoriasis [86]. Furthermore, viral antigens can increase recruitment of eosinophils [87].

The presence of HLA-A*3101 allele and homozygosity for epoxide hydrolase 1 (EPHX1) single nucleotide polymorphism rs1051740 (T-C) in exon 3, associated with modifications in epoxide hydrolase activity, were found in a patient with anticonvulsant HSR, possibly triggered by HHV reactivation, suggesting a genetic predisposition to HSR upon HHV reactivation [41].

HHV-6 reactivation can be further observed in the absence of an HSR and is primarily associated with hepatotoxicity. Phillips et al. were able to show with the help of transmission electron microscopy that full and empty virus capsids accumulate in the nucleus of hepatocytes, while viral particles bud out into the nuclear envelope and the cytoplasm [19]. Over the years, several drugs were used to treat HHV reactivations *per se*, as well as opportunistic infections in HSR patients with HHV reactivation, including idoxuridine [88], cytarabine [89], and vidarabine [90]. More recently, ganciclovir was used in a patient with HHV-6 and CMV reactivations [42]. Vancomycin was used to treat an opportunistic infection with *Staphylococcus aureus* in a DIHS patient with HHV-6 reactivation [44]. However, treatment against HHV-6 reactivation is often withheld out of fear of worsening the condition due to an increasing number of drugs that the patient is exposed to [26].

Drug-induced adverse reactions represent a concern for patients, clinicians, the pharmaceutical industry, and health providers. Interestingly, very little mention of HHV-6 reactivation is made in a comprehensive article discussing DILI, including acute liver failure in major DILI registries from Europe and the United States [91].

In conclusion, this paper provides a multifaceted assessment of drugs implicated in HSR that have been reported to induce HHV-6 reactivation. This information may facilitate the possible link between the diagnosis of SCAR and DILI, and HHV-6 reactivation.

## Figures and Tables

**Table 1:** Detailed analysis of select HSR cases with HHV-6 reactivation.

Diagnosed condition and patient characteristics	Preexisting medical conditions and previous drug exposure	Characteristics of HSR	HHV-6 reactivation characteristics	Status of other bacteria and viral reactivation	Treatment and symptoms resolution	Ref. no.
Carbamazepine DIHS 14-year-old Japanese male	Sodium valproate (600 mg/day) and carbamazepine (200 mg/day) for epilepsy Cefaclor (600 mg/day) for suspected bacterial infection	Fever after 17 days of carbamazepine Facial and body angioedema, generalized lymphadenopathy, mild hepatosplenomegaly, generalized erythema without erosion 11 days after first episode of fever WBC count 31.7 × 10^9^/L (eosinophils 11%, atypical lymphocytes 12.5%), RBC count 4.46 × 10^12^/L, hemoglobin 13.6 g/dL, platelet count 169 × 10^9^/L, C-reactive protein 1.8 mg/dL, total protein 5.2 g/dL, albumin 3.1 g/dL, AST 137 IU/L, ALT 202 IU/L, LDH 714 IU/L, blood urea nitrogen 10 mg/dL, and creatinine 0.69 mg/dL Transient hypogammaglobulinemia (IgG 649 mg/dL upon admission and 1169 mg/dL on day 26, similar trends for IgM and IgA)	Anti-HHV-6 IgG titers increased from 1 : 10 upon admission to 1 : 10240 23 days later Anti-HHV-6 IgM titers unchanged HHV-6 DNA copy numbers decreased from 3.5 × 10^12^ copies/10^6^ PBMCs on day 3 to 6.3 × 10^3^ copies/10^6^ PBMCs on day 46	Anti-HHV-7 IgG titers increased from 1 : 80 to 1 : 160 No changes in HHV-7 DNA copy numbers CMV, EBV, HSV, VZV, or parvovirus B19 serology negative	IV methylprednisolone (30 mg/kg) pulse therapy, followed by oral prednisolone (30 mg/day)	[35]

Carbamazepine DIHS 24-year-old Caucasian female	Carbamazepine (200 mg/day) for oligosymptomatic partial-complex seizures	Fever (38.6°C) with generalized lymphadenopathy and a maculopapular exanthema of the trunk and the perioral region after 6 weeks of carbamazepine WBC count 9.1 × 10^9^/L (19.7% atypical lymphocytes and 8.1% eosinophils) and elevated ALT (50 IU/L) *γ*-GTP (160 IU/L) and alkaline phosphatase (114 IU/L)	Anti-HHV-6 IgM titers positive Anti-HHV-6 IgG titers increased from 1 : 320 to 1 : 1280 within 25 days of hospitalization HHV-6 DNA detected in serum	EBV, CMV, HBC, HCV, HIV, *Toxoplasma gondii* or *Borrelia burgdorferi *serology negative	n/a Symptoms resolved spontaneously	[36]

Carbamazepine DIHS 24-year-old Japanese female	Carbamazepine for 32 days for agitation and emotional instability Cefcapene pivoxil hydrochloride administered for supposed cervical lymphadenitis	Low-grade fever and cervical lymph node swelling within 2 weeks of carbamazepine Pleomorphic erythema of the trunk and lower extremities within another week Fever (39.0°C–39.9°C) and dark purplish pleomorphic erythema over the trunk and extremities within next 10 days WBC count 14.3 × 10^9^/L (14.8% eosinophils), with severe liver dysfunction characterized by elevated AST (262 IU/L), ALT (423 IU/L), and LDH (827 IU/L) after 32 days of carbamazepine Inguinal lymph node swelling developed and edema in the lower limbs and face progressed Hepatic function worsened	Anti-HHV-6 IgG titer 1 : 80 and the IgM titer <1 : 10 at time of carbamazepine discontinuation Anti-HHV-6 IgG titers increased to 1 : 2560 and IgM titers became positive at 1 : 10	EBV, CMV, parvovirus B19 and antirubeola virus within normal ranges EBV and CMV serology negative	Not discussed	[37]

Carbamazepine DIHS 43-year-old Japanese female	Haloperidol, paroxetine hydrochloride, levomepromazine, amobarbital, bromovalerylurea chlorpromazine, and carbamazepine for anxiety and confusion	Generalized exudative erythema and fever (39.2°C) after 14 days of carbamazepine Increased WBC count, particularly atypical lymphocyte count Leukocytosis (27.5 × 10^9^/L) with atypical lymphocytosis (36%, 9.9 × 10^9^/L) and eosinophilia (4.5%, 0.88 × 10^9^/L) Hepatic dysfunction with elevated AST (78 IU/L), ALT (106 IU/L), LDH (873 IU/L), and *γ*-GTP (556 IU/L) Transient hypogammaglobulinemia	Anti-HHV-6 IgG titers increased from 1 : 10 on day 6 to 1 : 5120 on day 11 Anti-HHV-7 IgG titers increased from 1 : 80 to 1 : 160 HHV-6 DNA detected on day 9, but not on day 11	EBV, CMV, HSV, VZV and parvovirus B19 serology negative	IV methylprednisolone (1000 mg/day) pulse therapy for 3 days, followed by oral prednisolone (60 mg/day)	[38]

Carbamazepine DIHS 54-year-old Turkish female	Carbamazepine for epilepsy	Fever after 20 days of carbamazepine Maculopapular rash, erythroderma, exfoliative dermatitis, fever, facial, and genital edema, lymphadenopathy, hypereosinophilia, hypogammaglobulinemia, atypical lymphocytosis, leukocytosis and abnormal liver function tests	Anti-HHV-6 IgG 1 : 60 on day 7 and 1 : 1920 on day 14 HHV-6 DNA detected by PCR on day 14	Not specified	Systemic steroids, IV immunoglobulin for 30 days	[39]

Carbamazepine DIHS 17-year-old Turkish male	Carbamazepine for brain surgery	Fever after 12 days of carbamazepine Maculopapular rash, erythroderma, bullous lesion, hypereosinophilia, anemia, atypical lymphocytosis, abnormal liver function tests	Anti-HHV-6 IgG 1 : 80 on day 7 and 1 : 2560 on day 14 HHV-6 DNA detected by PCR on day 14	Not specified	Systemic steroids for 21 days	[39]

Carbamazepine DIHS 23-year-old Turkish female	Carbamazepine for epilepsy	Fever after 24 days of carbamazepine Erythroderma, exfoliative dermatitis, vasculitis (purpura), genital ulcer, lymphadenopathy, leukocytosis, hypereosinophilia, atypical lymphocytosis, abnormal liver function tests	Anti-HHV-6 IgG 1 : 20 on day 7 and 1 : 1280 on day 14 HHV-6 DNA not specified detected by PCR on day 14	Not specified	Systemic steroids for 30 days	[39]

Carbamazepine DIHS 29-year-old Turkish male	Carbamazepine for brain surgery	Fever after 22 days of carbamazepine Maculopapular rash, vasculitis (purpura), facial and genital edema, hypereosinophilia, leukocytosis, abnormal liver function tests, splenomegaly	Anti-HHV-6 IgG 1 : 20 on day 7 and 1 : 1280 on day 14 HHV-6 DNA detected by PCR on day 14	Not specified	Systemic steroids for 20 days	[39]

Carbamazepine DIHS 15-year-old Turkish female	Carbamazepine for epilepsy	Fever after 20 days of carbamazepine Erythroderma, lymphadenopathy, leukocytosis, hypereosinophilia, atypical lymphocytosis, increased in serum amylase (pancreatitis), abnormal liver function tests, lung pneumonia	Anti-HHV-6 IgG 1 : 30 on day 7 and 1 : 1920 on day 14 HHV-6 DNA detected by PCR on day 14	Not specified	Systemic steroids for 18 days	[39]

Carbamazepine DIHS accompanied by dermatologic changes and HHV-6 reactivation 14-year-old Japanese male	Carbamazepine (200 mg/day) for localization-related epilepsy	Fever (37.8°C) after 3 weeks of carbamazepine Liver dysfunction with AST 403 IU/L, ALT 549 IU/L and LDH 637 IU/L upon admission Erythroderma with edematous changes spread over the entire body WBC count 34.5 × 10^9^/L, with 19.5% atypical lymphocytes and 23.5% eosinophils prior to day 11 Liver dysfunction worsened with AST 1170 IU/L and ALT 700 IU/L on day 15 No cervical, axial, or inguinal lymphadenopathy detected	HHV-6 DNA (32000 copies/*μ*g of DNA) detected by real-time PCR and anti-HHV-6 antibodies isolated from PBMCs on day 19 Anti-HHV-6 IgG increased 5120-fold on day 25	HAV, HBV, CMV, EBV serology negative	Corticosteroids (1 mg/kg/day)	[40]

Carbamazepine DIHS possibly triggered by HHV-6 reactivation 12-year-old Italian female	Amoxicillin Sodium valproate for generalized epileptic seizure, replaced with carbamazepine	Fever (>39°C), cutaneous rash with mild face edema and moderate laterocervical lymphadenopathy after 5 weeks of carbamazepine Normal hemochrome with moderate lymphopenia (0.8 × 10^9^/L) and increased AST (200 IU/L), ALT (181 IU/L), and *γ*-GTP (116 IU/L)	Anti-HHV-6 IgG titers >1 : 128 HHV-6 and HHV-7 DNA detected	EBV, CMV, toxoplasma, *Bartonella* serologies, influenza, adenovirus, and respiratory syncytial virus serology negative	IV methylprednisolone (1 mg/kg) for 3 days, followed by oral methylprednisolone	[41]

Carbamazepine DIHS 48-year-old Japanese male	Carbamazepine (400 mg/day) for a psychiatric disease	High-grade fever and erythematous rash on his trunk after 43 days of carbamazepine Leukocytosis (9.3 × 10^9^/L (0% eosinophilia/13% atypical lymphocytosis)) Lymphadenopathy Severe liver dysfunction with highest ALT level 1859 IU/L Worsening of rash and liver dysfunction at time of EBV reactivation Thyroid dysfunction at time of HHV-6 and HHV-7 reactivation	Increase in HHV-6 viral load Dramatic increase in anti-HHV-6 IgG titers	Increase in EBV viral load after 9 days of hospitalization HHV-7 reactivation	None	[42]

Phenobarbital DIHS 68-year-old Japanese male	Phenobarbital (120 mg/day) for epileptic fits	Erythematous rash on chest and trunk, fever and malaise after 43 days of phenobarbital Leukocytosis (17.4 × 10^9^/L (6% eosinophilia/1% atypical lymphocytosis)) Lymphadenopathy Liver dysfunction with highest ALT level 323 IU/L Neurological symptoms developed in conjunction with increased HHV-6 viral load Neurological symptoms reappeared with rise in HHV-7 viral load	HHV-6 DNA detected 19 days after hospitalization Dramatic rise in anti-HHV-6 IgG titers but not IgM titers	EBV viral load increased as HHV-6 decreased HHV-7 viral load increased within a few months of HHV-6 CMV viral load increased with no clinical symptoms	Prednisolone (60 mg/day)	[42]

Salazosulfapyridine DIHS 68-year-old Japanese male	Salazosulfapyridine (2000 mg/day) for rheumatoid arthritis Previous exposure to prednisolone	High-grade fever, malaise, and rash extending to trunk and lower extremities after 28 days of salazosulfapyridine Leukocytosis (17.7 × 10^9^/L (0% eosinophilia/2% atypical lymphocytosis)) Facial edema, lymphadenopathy and leucopenia upon admission Slight liver dysfunction with highest ALT level 44 IU/L Herpes labialis with herpetic stomatitis at time of HSV reactivation 13 days after admission Erythematous rash with transient high fever and dry cough developed at time of CMV antigens detection	HHV-6 DNA detected in blood upon admission Dramatic rise in anti-HHV-6 IgG titers	HSV reactivation HSV antigens EBV and CMV DNA detected	Prednisolone (40 mg/day)	[42]

Mexiletine DIHS 74-year-old Japanese male	Mexiletine for arrhythmia	High-grade fever and generalized erythematous rash on the chest and trunk after 31 days of mexiletine Leukocytosis (9.2 × 10^9^/L (14% eosinophilia/2% atypical lymphocytosis)) Lymphadenopathy Liver dysfunction with highest ALT level 156 IU/L Cytomegalovirus antigenemia and massive internal bleeding following detection of CMV DNA Slight liver dysfunction following detection of EBV DNA	HHV-6 DNA detected Increased anti-HHV-6 IgG titers	Increased EBV and CMV viral loads	Prednisolone (50 mg/day) Ganciclovir (200 mg/day)	[42]

Anticonvulsant DIHS with HHV-6 reactivation developed after initial exanthema subitum complicated with febrile seizure 11-month-old Japanese female	IV diazepam for right tonic hemiconvulsions and upward deviation of the eyes Oral carbamazepine initiated in place of thiopental 10 days after hospitalization Carbamazepine replaced with phenobarbital IV phenytoin added on day 14 and continued for 11 days	Fever (39.1°C) without skin symptoms after 14 days of phenobarbital Erythematous rash appeared and spread over the entire body after 4 additional days Maculopapular erythematous rash appeared on face, extremities, and trunk after carbamazepine exposure Cervical lymphadenopathy Facial edema after phenobarbital discontinuation Elevated eosinophil count (1.9 × 10^9^/L), AST (105 IU/L) and ALT (45 IU/L)	HHV-6 DNA first detected on day 5 (635 copies/mL in serum and 31.5 copies/mL in cerebrospinal fluid), coinciding with the eruptive stage of exanthema subitum HHV-6 DNA undetectable on day 16 HHV-6 DNA detected on day 28 (805 copies/mL in serum), coinciding with high fever and generalized erythema HHV-6 DNA increased on day 30 (4360 copies/mL in serum) when DIHS was suspected Anti-HHV-6 IgG titers negative upon admission Anti-HHV-6 IgG titers elevated on day 11 (256-fold) and day 34 (128-fold)	Not specified	Continuous infusion of thiopental and assisted mechanical ventilation, as well as IV methylprednisolone pulse therapy (30 m/kg/day for 3 days), *γ*-globulin, and acyclovir therapy for initial condition IV methylprednisolone for resolution of DIHS	[43]

Phenobarbital DIHS and fulminant hemophagocytic syndrome associated with HHV-6 reactivation 25-year-old Laotian female	Oral phenobarbital for epileptic seizures	Fever (40°C), diffuse pruritic exfoliative dermatitis with edema of the face and purpuric lesions on the extremities, enlarged cervical, axillary and inguinal lymph nodes, and hepatomegaly after 18 days of phenobarbital Atypical lymphocytes with leukocytosis (24.5 × 10^9^/L), eosinophilia (5.14 × 10^9^/L), and lymphocytosis (7.1 × 10^9^/L) with predominant T phenotype (90% CD3, 1% CD19) Liver dysfunction with elevated ALT (465 IU/L), AST (165 IU/L), and LDH (19000 U/mL) Severe erythroderma complicated by methicillin-resistant *Staphylococcus aureus* septicemia on day 13 Acute hepatic failure with ALT 1415 IU/L and AST 2525 IU/L	Anti-HHV-6 IgG titers 1 : 80 on day 14 and >1 : 320 on day 29 HHV-6 DNA not detected in serum by PCR	EBV, CMV, HIV, human T-cell lymphotropic virus type 1, parvovirus B19, HCV, HBV, picornavirus, *Toxoplasma gondii* and *Treponema pallidum* serology negative	Oral corticosteroids and etoposide Corticosteroid therapy continued >1 year IV vancomycin (2 g/day)	[44]

Phenobarbital DIHS with multiple viral reactivations 43-year-old Japanese male	Phenobarbital (300 mg daily) for a brain aneurysm	High-grade fever, bilateral cervical and inguinal lymphadenopathy, and hepatomegaly after 44 days of phenobarbital Blood cell count 11.8 × 10^9^/L, with eosinophil count 3.08 × 10^9^/L Liver dysfunction with elevated AST 273 IU/L, ALT 770 IU/L, LDH 582 IU/L and C-reactive protein 12.7 mg/dL Hypogammaglobulinemia with serum IgG levels 769 mg/dL, IgA levels 47 mg/d/L and IgM levels 76 mg/dL Skin biopsy revealed dense infiltration consisting mainly of mononuclear cells and eosinophils in the upper dermis	Dramatic increases in anti-HHV-6 IgG on day 19	Dramatic increases in anti-CMV IgM on day 19 Increase in anti-HHV-7 IgG EBV serology negative	Initial HSR symptoms resolved spontaneously Treatment not administered	[45]

Phenobarbital DIHS 31-year-old Japanese female	Phenobarbital for depression	Fever, severe systemic erythematous eruptions followed by systemic lymphadenopathy and hepatosplenomegaly after 3 weeks of phenobarbital WBC count 105.4 × 10^9^/L, with 25% eosinophils and 52% atypical lymphocytes, platelet count 161 × 10^9^/L and hemoglobin concentration 9.4 g/dL Liver dysfunction with AST 70 IU/L, ALT 93 IU/L, alkaline phosphatase 824 IU/L, *γ*-GTP 592 IU/L, and LDH 364 IU/L	Anti-HHV-6 IgG 1 : 2560 and IgM 1 : 40 HHV-6 DNA 6.3 × 10^3^ copies/mL in PBMCs	EBV, CMV, VZV, and HSV serology negative	Methylprednisolone (1000 mg/day) for 3 days, followed by prednisolone (60 mg/day)	[46]

Aromatic anticonvulsants DIHS developed concomitantly to HHV-6 seroconversion in 56-year-old Japanese male	Hypertension Sodium valproate (600 mg/day) for a 6-month history of generalized convulsion of unknown cause Phenytoin (134 mg/day) and phenobarbital (66 mg/day) for 3 weeks Acyclovir (20 mg/kg/day) for was believed to be HSV encephalitis	Generalized erythematous macules after 3 weeks of phenytoin and phenobarbital High-grade fever (39.8°C), generalized erythroderma and edema within 1 week WBC count 23.3 × 10^9^/L (23% eosinophils and 9.5% atypical lymphocytes) Abnormal liver function Skin biopsy revealed a spongiotic epidermis with liquefactive degeneration of the epidermal basal layer Encephalopathy of nonmetabolic origins Death with multiple organ failure 18 days after falling into a coma and 5 weeks after rash appearance	Anti-HHV-6 IgG titers increased from 1 : 80 one month after appearance of macular rash to 1 : 640 within next 2 weeks Anti-HHV-6 IgM negative	HSV-1, HSV-2, VZV, CMV, EBV, measles, rubella, and mumps serology positive	Oral prednisone (30 mg/day) IV diazepam for frequent partial seizures developed during hospital stay	[47]

Anticonvulsant DIHS 6-year-old Tunisian child	Sodium valproate (600 mg/day) intermittently for 41 days Ethosuximide (500 mg/day) intermittently for 29 days for epileptic absences Ethosuximide interrupted prior to the hospitalization	High-fever (40°C), facial edema, diffuse pruritic morbilliform skin eruption with vesicular, and target lesions after 41 days of intermittent sodium valproate Leukocytosis of 17.9 × 10^9^/L, eosinophilia of 2.0 × 10^9^/L, lymphocytosis of 8.95 × 10^9^/L and monocytosis of 1.43 × 10^9^/L, with normal platelet count Elevated C-reactive protein (57 mg/mL), AST (70 IU/L), *γ*-GTP (50 IU/L), and LDH (1020 IU/L) Histologic analysis revealed a spongiotic epidermis with necrotic keratinocytes and a lymphocytic perivascular dermal infiltrate with a few hyperchromatic lymphocytes with irregular nuclei	Anti-HHV-6 IgG titers increased from 1 : 40 to 1 : 120 in the course of 120 days	EBV, HIV, HAV, HBV, HCV, CMV, parvovirus B19, herpes simplex virus and* Mycoplasma pneumoniae* serology negative	Oral prednisone (1 mg/kg/day)	[48]

Zonisamide DIHS 29-year-old Japanese male	Zonisamide (300 mg/day) for temporal epilepsy, interrupted 7 days prior to admission Cefcapene pivoxil hydrochloride hydrate, acetaminophen, tranexamic acid, and lansoprazole prescribed 7 days prior to admission	Acute kidney injury and diffuse skin rash with edema of the face after 2 months of zonisamide Fever, elevated AST (65 IU/L), ALT (132 IU/L), and *γ*-GTP (153 IU/L) levels 4 days prior to admission Facial edema and a generalized pruritic maculopapular rash with lichen upon admission Lymphadenopathy, eosinophilia, renal dysfunction, liver dysfunction, and hypogammaglobulinemia Splenomegaly and enlarged kidneys with serum creatinine level of 8.20 mg/dL Fever persisted and eruptions evolved to flaccid vesicles and bullae 4 days after admission Skin biopsy revealed multiple epidermal bullous formation with eosinophilic abscess Renal biopsy performed revealed glomeruli with minor abnormality, with infiltration of mononuclear cells and eosinophilic cells around each glomerulus and focal interstitium and interstitial edema with swelling and degeneration of tubular epithelial cells	HHV-6 DNA detected by PCR on day 6 but not on day 20 Anti-HHV-6 IgG levels increased from 1 : 16 on day 2 to 1 : 256 on day 27	Not specified	Hemodialysis Prednisolone (60 mg/day) started 4 days after admission	[49]

Lamotrigine DIHS 26 year-old Italian female	Additive mastoplasty for von Willembrandt disease in childhood Current escitalopram and lamotrigine for a bipolar disease Cefixime (400 mg twice/day) for 3 days, betamethasone, 1 mg and fexofenadine 120 mg for 1 day, and azithromycin 500 mg once/day for 3 days for fever (≤39.5°C) developed 16 days prior to admittance	Fever after 30 days of lamotrigine Asthenia, nausea, myalgia, and arthralgia, as well as a maculopapular rash on the face, neck, trunk and superior and inferior limbs, hard and tender lymph nodes, and hepatomegaly upon admission Lymphadenopathy Eosinophilic leukocytosis (31.7 × 10^9^/L, with 18% eosinophils) Increases in CD4 (3.7 × 10^9^/L) and CD8 (5.8 × 10^9^/L) T lymphocyte counts Severe liver dysfunction with 19-fold increased ALT and 14-fold increased AST High bilirubin level (2.84 mg/dL) and low prothrombin activity (39%)	Anti-HHV-6 IgG and IgM detected on day 11 HHV-6 DNA 8590 copies/mL in blood with real-time PCR	HAV, HBV, HCV, EBV, CMV, rubella, adenovirus, coxsackievirus, influenza virus A/B, parainfluenza virus serology negative Antibodies against *Borrelia burgdorferi*, *Rickettsia conori*, *Rickettsia typhi*, *Chlamydia trachomatis,* and *Leishmania infantum* negative	IV betamethasone (8 mg 3 times/day) and IV acyclovir (250 mg 3 times/day) for 8 days Fresh frozen plasma infusions, physiological solutions, and proton pomp inhibitors Prednisone (50 mg/day)	[50]

Lamotrigine DRESS 75-year-old French male	Lamotrigine (25 mg/day) for generalized tonic-clonic seizures	Diffuse exanthematous maculopapular rash affecting between 50% and 70% of the skin surface, fever, peripheral lymphadenopathies, and abdominal pain after 40 days of lamotrigine Hyperleukocytosis with hypereosinophilia Skin biopsy showed marked infiltration of the dermo-epidermal junction with lymphocytes and keratocyte necrosis Acute edematous pancreatitis diagnosed based on increased pancreas size Bilateral basal crackles on lung auscultation and hepatomegaly, as well as severe acute cytolytic and cholestatic hepatitis with hepatocellular insufficiency Oliguria and kidney failure Multiorgan failure by day 55	Positive anti-HHV-6 IgM serology HHV-6 DNA 11000 copies/mL	HIV, HAV, HBV, HCV, EBV, HSV-1 HSV-2, CMV serology negative	Prednisone (1 mg/kg/day) for 20 days	[51]

Carbamazepine DIHS 59-year-old Japanese male	Carbamazepine	Fever (>38.5°C) after 30 days of carbamazepine Liver dysfunction with ALT 119 IU/L and *γ*-GTP 79 IU/L WBC count 16.2 × 10^9^/L and eosinophils count 0.45 × 10^9^/L (16% atypical lymphocytes) Lymph node enlargement	Anti-HHV-6 IgG titers 1 : 160 during active phase and 1 : 1280 during recovery phase	Not specified	Prednisolone (40 mg/day)	[52]

Carbamazepine DIHS 56-year-old Japanese female	Carbamazepine	Fever (>38.5°C) after 22 days of carbamazepine Severe liver dysfunction with ALT 706 IU/L and *γ*-GTP 318 IU/L WBC count 11.5 × 10^9^/L and eosinophils count 0.83 × 10^9^/L (15% atypical lymphocytes) Lymph node enlargement	Anti-HHV-6 IgG titers 1 : 160 during active phase and 1 : 160 during recovery phase	Not specified	Prednisolone (40 mg/day)	[52]

Carbamazepine DIHS 62-year-old Japanese female	Carbamazepine	Fever (>38.5°C) after 51 days of carbamazepine No liver dysfunction with ALT 9 IU/L and *γ*-GTP 40 IU/L WBC count 7.8 × 10^9^/L and eosinophils count 1.2 × 10^9^/L (8% atypical lymphocytes) Lymph node enlargement	Anti-HHV-6 IgG titers 1 : 10 during active phase and 1 : 640 during recovery phase	Not specified	Betamethasone (2 mg/day)	[52]

Carbamazepine DIHS 53-year-old Japanese female	Carbamazepine	Fever (>38.5°C) after 26 days of carbamazepine Liver dysfunction with ALT 327 IU/L and *γ*-GTP 108 IU/L WBC count 6.4 × 10^9^/L and eosinophils count 0.39 × 10^9^/L Lymph node enlargement	Anti-HHV-6 IgG titers 1 : 640 during active phase and 1 : 10240 during recovery phase	Not specified	Corticosteroids not used	[52]

Carbamazepine DIHS 71-year-old Japanese male	Carbamazepine	Fever (>38.5°C) after 50 days of carbamazepine Liver dysfunction with ALT 108 IU/L and *γ*-GTP 140 IU/L WBC count 8.1 × 10^9^/L and eosinophils count 0.50 × 10^9^/L Lymph node enlargement	Anti-HHV-6 IgG titers 1 : 10 during active phase and 1 : 160 during recovery phase	Not specified	Prednisolone (30 mg/day)	[52]

Zonisamide DIHS 27-year-old Japanese female	Zonisamide	Fever (>38.5°C) after 26 days of zonisamide Severe liver dysfunction with ALT 401 IU/L and *γ*-GTP 220 IU/L WBC count 21.3 × 10^9^/L and eosinophils count 0.66 × 10^9^/L (49% atypical lymphocytes) Lymph node enlargement	Anti-HHV-6 IgG titers 1 : 40 during active phase and 1 : 1280 during recovery phase	Not specified	Prednisolone (30 mg/day)	[52]

Phenobarbital SJS 72-year-old Japanese female	Phenobarbital	Fever (>38.5°C) after 31 days of phenobarbital Mild liver dysfunction with ALT 72 IU/L and *γ*-GTP 28 IU/L WBC count 15.8 × 10^9^/L and eosinophils count 0.42 × 10^9^/L 5% detachment of the total body surface area	Anti-HHV-6 IgG titers 1 : 40 during active phase and 1 : 20 during recovery phase	Not specified	Prednisolone (40 mg/day)	[52]

Phenytoin SJS 19-year-old Japanese female	Phenytoin	Fever (>38.5°C) after 26 days of phenytoin Liver dysfunction with ALT 90 IU/L and *γ*-GTP 436 IU/L WBC count 12.8 × 10^9^/L and eosinophils count 0.90 × 10^9^/L Lymph node enlargement 10% detachment of the total body surface area	Anti-HHV-6 IgG titers 1 : 80 during active phase and 1 : 80 during recovery phase	Not specified	Prednisolone (60 mg/day)	[52]

Zonisamide TEN 71-year-old Japanese male	Zonisamide (300 mg/day) for symptomatic epilepsy History of long-term valproate sodium therapy	Fever (40.2°C) and rash after 23 days of zonisamide Erythematous macules resulting in diffuse areas of erythema with erosions and blisters located on the trunk and upper extremities 40% detachment of the total body surface area, with extensive hemorrhagic erosions on the lips, oral mucosa, pharynx, and larynx Elevated ALT (48 IU/L), LDH (379 IU/L), *γ*-GTP (111 IU/L), and C-reactive protein (11.3 mg/L) WBC count 12.5 × 10^9^/L and eosinophils count 1.02 × 10^9^/L Skin biopsy revealed prominent eosinophilic necrosis of the keratinocytes and subepidermal blister Moderate inflammatory infiltrate consisting of mononuclear cells in the upper dermis Skin eruptions and high fever returned 9 days after initial onset	Anti-HHV-6 IgG titers 1 : 10 during active phase (day 4) and 1 : 1280 during recovery phase (day 22) HHV-6 DNA levels increased from 2.0 × 10^1^ copies/10^6^ cells on day 4 to 1.3 × 10^2^ copies/10^6^ cells on day 22 in PBMCs	No change in HSV, CMV or EBV IgG titers	IV immunoglobulin therapy (5 g/day) for 3 days resulted in slowing of disease progress	[52]

Zonisamide TEN 66-year-old Japanese male	Zonisamide	Fever (>38.5°C) after 25 days of zonisamide Mild liver dysfunction with ALT 58 IU/L and *γ*-GTP 61 IU/L WBC count 10.1 × 10^9^/L and eosinophils count 0.28 × 10^9^/L 40% detachment of the total body surface area	Anti-HHV-6 IgG titers 1 : 40 during active phase and 1 : 40 during recovery phase	Not specified	Methylprednisolone (500 mg/day), followed by Prednisolone (30 mg/day)	[52]

Normal ranges: ALT 9–56 IU/L, AST 14–56 IU/L, *γ*-GTP 4–68 IU/L, LDH 116–250 IU/L, alkaline phosphatase 108 IU/L [35, 36, 38, 40, 44, 45, 52, 53], WBC 3.3 × 10^9^/L–8.6 × 10^9^/L, eosinophil count 0.07 × 10^9^/L–0.45 × 10^9^/L (eosinophils <8% of total circulating leukocytes), lymphocyte count 2.5 × 10^9^/L–5.5 × 10^9^/L, monocyte count <1 × 10^9^/L [48, 52, 53], IgG 778–1794 mg/dL, IgA 80–413 mg/dL, IgM normal 37–254 mg/dL [45].

Abbreviations: ALT: alanine aminotransferase; AST: aspartate aminotransferase; CMV: cytomegalovirus; EBV: Epstein-Barr virus; HAV: hepatitis A virus; HBV: hepatitis B virus; HCV: hepatitis C virus; HHV: human herpes virus; HIV: human immunodeficiency virus; HSV: human herpes simplex virus; IV: intravenous; LDH: lactate dehydrogenase; PBMC: peripheral blood mononuclear cell; PCR: polymerase chain reaction; RBC: red blood cell; SJS: Stevens-Johnson syndrome; TEN: toxic epidermal necrolysis; VZV: varicella-zoster virus; WBC: white blood cell; *γ*-GTP: gamma-glutamyl transpeptidase.

## Abbreviations

ADR: Adverse drug reaction
ALT: Alanine aminotransferase
AST: Aspartate aminotransferase
CIHHV-6: Chromosomally integrated human herpes virus-6
CMV: Cytomegalovirus
DIHS: Drug-induced hypersensitivity syndrome
DILI: Drug-induced liver injury
DRESS: Drug reaction with eosinophilia and systemic symptom
EBV: Epstein-Barr virus
HAV: Hepatitis A virus
HBV: Hepatitis B virus
HCV: Hepatitis C virus
HHV: Human herpes virus
HIV: Human immunodeficiency virus
HLA: Human leukocyte antigen
HSR: Hypersensitivity reaction
HSV: Human herpes simplex virus
IFN: Interferon
IL: Interleukin
IV: Intravenous
LDH: Lactate dehydrogenase
PBMC: Peripheral blood mononuclear cell
PCR: Polymerase chain reaction
SCAR: Severe cutaneous adverse reactions
SJS: Stevens-Johnson syndrome
TEN: Toxic epidermal necrolysis
TNF: Tumor necrosis factor
VZV: Varicella-zoster virus
*γ*-GTP: Gamma-glutamyl transpeptidase.
